# Features of Thermomechanical Stability of Anionic–Cation Exchange Matrix “Polikon AC” on Viscose Non-Woven Materials

**DOI:** 10.3390/membranes11100734

**Published:** 2021-09-27

**Authors:** Denis Terin, Marina Kardash, Sergey Korchagin, Sergey Tsyplyayev, Vladimir Cherkasov, Tamara Druzhinina

**Affiliations:** 1 Yuri Gagarin State Technical University of Saratov, 77 Polytechnicheskaya St., 410008 Saratov, Russia; terinden@mail.ru (D.T.); tsiplyayev@mail.ru (S.T.); 2 Saratov State University, 83 Astrakhanskaya St., 410012 Saratov, Russia; 3 MIREA–Russian Technological University, 78 Vernadsky Avenue, 119454 Moscow, Russia; 4 Financial University under the Government of the Russian Federation, 4th Veshnyakovsky Proezd, 4, 109456 Moscow, Russia; SAKorchagin@fa.ru; 5 National University of Science and Technology “MISiS”, 4 Leninskiy Prospekt, 119991 Moscow, Russia; v.basenchikov@yandex.ru; 6 Moscow State University of Design and Technology, 33 Sadovnicheskaya St., 117997 Moscow, Russia; TamaraDruzhinina@mail.ru

**Keywords:** bifunctional cation exchanger, big data, composite heterogeneous materials, molecular model-ing, polyfunctional anion exchanger, semantic analysis, thermogravimetry-differential thermal analysis, viscose fiber

## Abstract

The thermomechanical stability of the anion–cation exchange matrix “Polikon AC” on viscose nonwoven materials is investigated. In this work, a molecular model of a solvation environment for experimentally obtained “Polikon AC” mosaic membranes is refined. Mosaic membranes on a viscose fiber base were fabricated by the method of polycondensation filling. The temperature dependence of deformation was investigated for dry and wet anion and cation exchange membrane components at a constant tensile load of 1.5 N and a heating rate of 8 °C/min. The effect of moisture content on the deformation of anionite and cationite fragments under a constant external tensile load of 1.5 and 3 N in a temperature range up to 100 °C was studied.

## 1. Introduction

**Figure 1:**
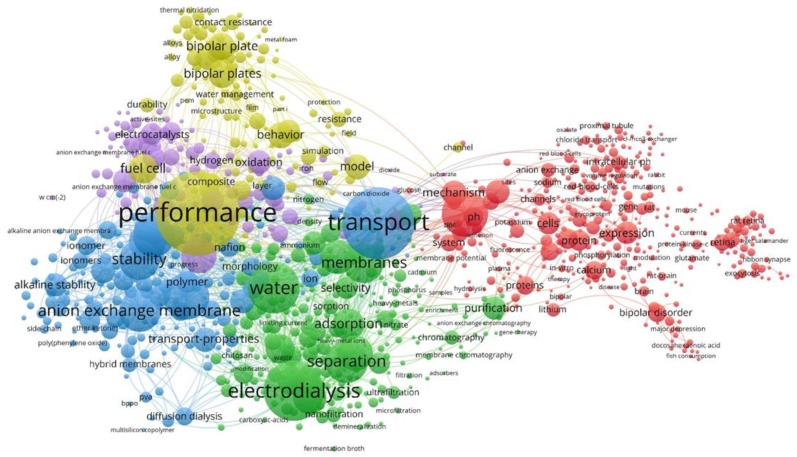
Semantic analysis of the most common key concepts.

Today, it is undeniable that the creation of synthetic ion-exchange membranes is possible only at the junction of macromolecular chemistry, quantum chemical calculations, electrochemistry, thermodynamics, ion exchange, colloid chemistry, and modern research methods [1]. Water shortages and access to clean water are serious problems throughout the world, directly affecting one-fifth of the world’s population [2]. At the same time, the desalination of seawater is becoming one of the mass sources of drinking water and is important for ensuring a sustainable quality of life while meeting high world environmental standards. This includes the treatment of urban and agricultural waste and the treatment of industrial effluents. The growing need for safe drinking water, the purification of wastewater from small molecular fragments of pollutants, and the recovery of valuable products has determined the importance of membrane separation processes as one of the fastest-growing technologies. Membrane technology is recognized as energy efficient, easily scalable, and clean, with a large prospective expansion of its application to the growing need for implementing more stable industrial processes [3]. For this work, a set of bibliographic data was studied, formed on the literature. The generated data set was processed using software for visualization and analysis of the related data [4], which made it possible to form semantic network cartograms (Figure 1).

Visualization in the Network Visualization mode includes the keywords and phrases most often used in sources. The frequency of occurrence of a keyword (phrase) in research is estimated by the size of the “cloud” in which the term of interest is enclosed. Cloud color differentiation allows the identification of clusters of similar elements and subsets. This analysis allowed us to detect five clustered systems linking a practical application, the structural and operating characteristics, methods of modification, ion transport mechanisms, and the model used to describe them. The proposed approach makes it possible to quickly identify the main trends, substantiate the relevance of the planned research, and graphically present the dynamics and intensity of interest in the problem for the selected period.

**Figure 2:**
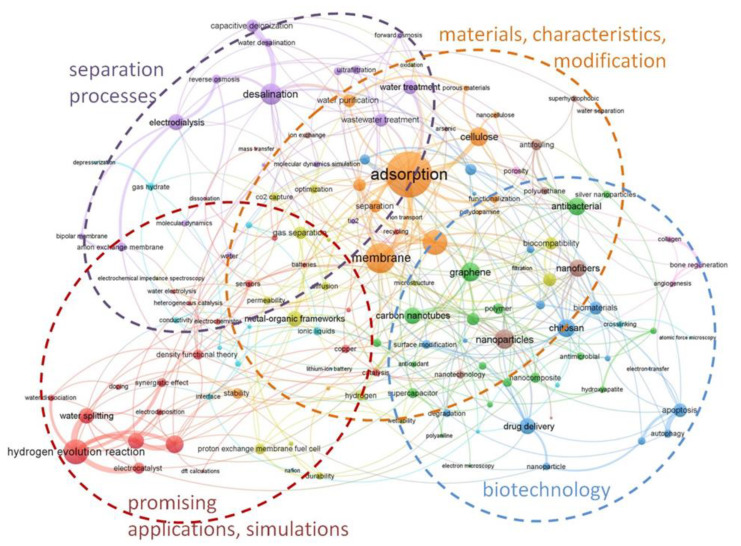
Membrane science map 2021. A co-occurrence network built using words found in titles and excerpts from documents published in 2021 (January to July).

A search of the WoS scientific publications database using the Advanced Search Query Builder identified 7527 articles in 2021 (January to July) that, according to the terminology, could be associated with the keywords: “membrane (abstract, title), ionic transfer, or ion exchange or membrane materials”; this speaks of the rapidly developing scientific field of membrane materials science. A semantic analysis (Figure 2) of the articles made it possible to identify the four most promising areas of research related to separation processes and materials technologies, their characteristics, prospects for practical use, and modeling.

The fabrication of multilayer composite heterogeneous anion–cation exchange membranes of a mosaic structure, promising for use in sorption and electro membrane processes, especially for the capacitive deionization of water, has been a very urgent task in the last decade [5]. Multilayer cellulose-based membranes are successfully used for gas separation, pervaporation, nanofiltration, and reverse osmosis. Sedelkin et al. investigated the mechanism of transport of ions in the pores of the semipermeable diacetate cellulose membranes in the ultrafiltration aqueous solutions containing serum proteins and mineral substances representing ions Cl^−^, K^+^, Ca^2+^, Na^+^ [6]. A model of ion transport in the capillary pores of ultrafiltration membranes is proposed.

The work [7] of the same research group determined the effect of vapors of aqueous–organic mixtures used to modify powdered cellulose diacetates in the structure and properties of the resulting polymer membranes and also experimentally investigated [8] the structure and properties of cellulose diacetate filtration membranes with solid fillers, used for the heat-treated waste of millet threshing. The porometric parameters of the starting polymer and solid fillers, the rheological properties of the molding solutions, and the performance characteristics of the resulting composite membranes were determined.

Vorotyntsev et al. determined the mechanism of transmembrane transport of ammonia and water through a cellulose acetate membrane [9]. Sorption of water and ammonia was realized and localized in the active centers of the polymer matrix due to the formation of hydrogen bonds, with both hydroxyl groups and the oxygen of the ester groups of cellulose acetate. The possibility of using cellulose acetate membranes as a matrix for solid-phase luminescent determination of pyrene in aqueous micellar media was shown in the article [10].

Lazarev et al. presented the results of studying the state of water in the composite acetate cellulose membrane MGA-95; water plays the role of a plasticizer and structures the macromolecules of the amorphous phase of the acetate cellulose membrane and transfers it to the liquid crystalline phase, forming additional capillary spaces [11].

Anokhina et al. prepared composite cellulose membranes by casting a solution of cellulose in N-methylmorpholine-N-oxide on a nonwoven polyester substrate [12]. The membranes were studied in the process of the nanofiltration of aprotic solvents. Sorption experiments showed a noticeable difference in the interaction of individual solvents with the membrane material: a low degree of cellulose swelling was found in tetrahydrofuran (THF, 37%) and a higher degree in dimethylsulfoxide (DMSO, 230%). In addition, it was found that the coefficient of retention of solutes by composite membranes correlates with the degree of swelling of cellulose. Anokhina et al. investigated the process of dissolution of cellulose in [Emim]OAc ionic liquid with the addition of a co-solvent, DMSO, and analyzed the possibility of creating composite cellulose membranes for the process of nanofiltration of organic media [13]. Hybrid membranes containing cubic and tetragonal MOF-5 structures were investigated [14]. They were synthesized, characterized, and included in a matrix of cellulose acetate.

Malakhov and coworkers described the use of nanocellulose to improve the transport properties of ultrafiltration membranes used for the purification of aqueous media [15]. The effect of the nature of the precipitant on the nanofiltration characteristics of cellulose membranes obtained from solutions in 1-ethyl-3-methylimidazolium acetate or a mixture of this ionic liquid with dimethyl sulfoxide was studied [16]. The best nanofiltration characteristics were demonstrated by a cellulose membrane obtained by precipitation in an aqueous solution (30%) of acetic acid: PDMF = 0.67 kg/m^2^ h atm. Syrtsova et al. proposed a “green” method for obtaining new composite membranes from a cellulose solution in phosphoric acid on various ultrafiltration substrates [17]. In terms of “green” chemistry, cellulose is the most promising polymer because it has a constantly renewable plant raw material base and biodegrades under natural conditions. This method can be used for industrial applications and differs from the traditional viscose method for producing cellophane and other known methods for producing cellulose gas separation membranes in the absence of gaseous emissions and wastewater. The proposed method of obtaining membranes allows one to achieve the uniform deposition of dense gas separation layers of cellulose, while the membranes demonstrate a level of gas permeability three orders of magnitude higher than the level of gas permeability of cellophane films. It was noted that the highest ideal selectivity was achieved for membranes with a cellulose gas separation layer on viscose substrates.

Aziz et al. studied the advances made in the chemical modification of cellulose nanocrystals (CNCs) and their corresponding applications. Different synthetic methods such as esterification and etherification, cross-linking, and grafting techniques and parameters are used in cellulose nanocrystals formation [18,19]. Additionally, Aziz et al. evaluated nanoparticles that were uniformly dispersed in a bio-based epoxy resin and the mechanical properties, adhesive strength, and morphology of the resultant nanocomposites [20].

We have previously shown [21,22] the possibility of using a nonwoven material made of viscose fiber to obtain a polymeric composite material “Polikon”, obtained by the method of the polycondensation filling of polymer composites by synthesizing and curing a weakly basic anion exchanger or a strongly acidic sulfonic cation exchanger on the surface and in the structure of a nonwoven material made of viscose fiber. We have developed the techniques and technological parameters of the processes of their preparation and have also assumed the bipolar nature of ion transfer in such membranes.

The review [1] examines the general understanding of the technology of the synthesis of bipolar membranes, describing the current state of affairs in the field of membrane synthesis, properties, theoretical models, and applications based on scientific publications and patents in this area over the past 70 years. Optimized membrane properties are discussed to achieve targeted optimized performance; new applications are presented, including a list of the shortcomings of existing membranes that need to be overcome to discover promising new applications.

The purpose of this work is the experimental testing of mathematical models described by us earlier, which allow us to relate the production conditions and technological parameters to the physicomechanical characteristics of the obtained “Polikon” mosaic membranes. Additionally, we discuss the expansion of ideas about the polycondensation processes of synthesis and the structure formation of the anion–cathine exchange matrix on the surface and structure of chemical fibers, the establishment of the main regularities of their implementation in a single technological cycle, and the possibility of modification to improve the operational characteristics of the developed materials.

By its nature, polycondensation filling consists of the covalent grafting of hydrophilic polymer fragments onto a promising viscose matrix/fibrous base to expand the range of new types of membrane systems with useful properties such as high surface area, mechanical strength, thermal stability, and low coefficient of thermal expansion. This work is devoted to the refinement of the research paradigm in the field of the synthesis and targeted modification of the structure and properties of the heterogeneous anion–cation exchange mosaic membrane “Polikon” and the search for new areas of practical application.

## 2. Materials, Methods, and Models

**Figure 3:**
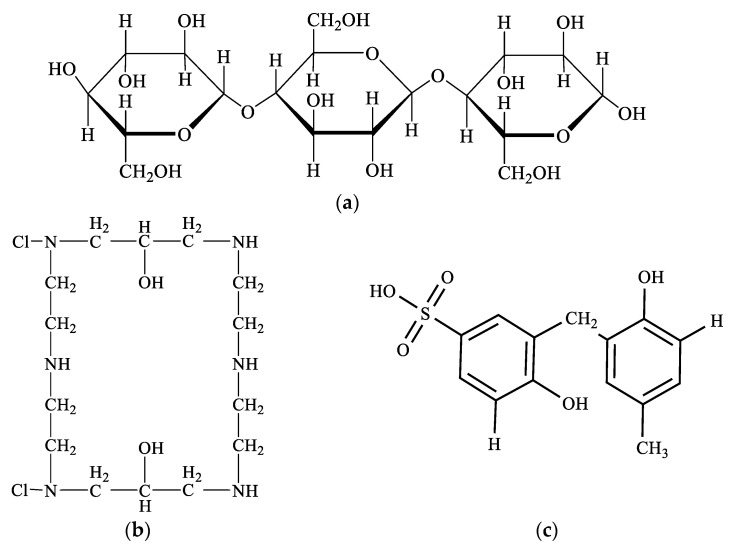
Elemental constituents of the membrane fragments: (**a**) fibrous base, (**b**) anion exchanger, and (**c**) cation exchanger.

The initial components of the anion–cation exchange membrane “Polikon AC” are a fibrous base—a viscose nonwoven material (according to GOST ISO 2076-2015) (Figure 3a), on the surface and in the structure of which a polyfunctional anion exchanger of mixed basicity is synthesized and formed. It contains secondary and tertiary amino groups and quaternary ammonium groups (obtained from polyethylene polyamines (according to TU 6-02-594-85) and epichlorohydrins (according to GOST 12844-74); Figure 3b) and a bifunctional cation exchanger with ion-exchange groups –SO_3_H and –OH (obtained from n-phenol sulfonic acid and formaldehyde; Figure 3c). Figure 2 shows elementary-optimized fragments of a viscose nonwoven fabric (a), anionite (b), and cationite matrices (c).

**Figure 4:**
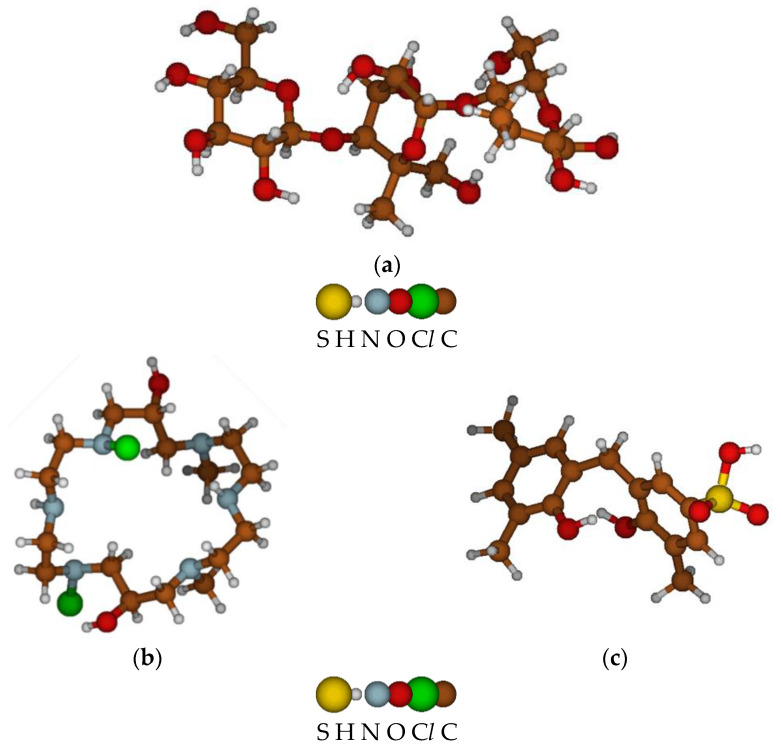
Elementary optimized fragments of viscose nonwoven fabric (**a**), anionite (**b**), and cationite (**c**) matrices.

The minimum block geometric volume in Å was determined, occupied by elementary viscose (6.94 × 6.41 × 15.31), anionite (9.28 × 6.01 × 9.89), and cationite (6.76 × 6.85 × 14.46) fragments. The model of a viscose microfibril contains 71 atoms (Figure 4a), has a total grid surface area of ~496.124 Å^2^, and an occupied molecular volume of 427.2 Å^3^, with a total mass of ~516.497 amu. The anionite fragment has 62 atoms (Figure 4b), with a total surface area of ~410.329 Å^2^ occupied by a molecular volume of 388.590 Å^3^, with a mass of 415.407 amu. The cation-exchange fragment consists of 40 atoms (Figure 4c), has a total grid surface area of ~324.572 Å^2^, occupies a molecular volume of 276.806 Å^3^, and has a total mass of ~322.376 amu.

**Figure 5:**
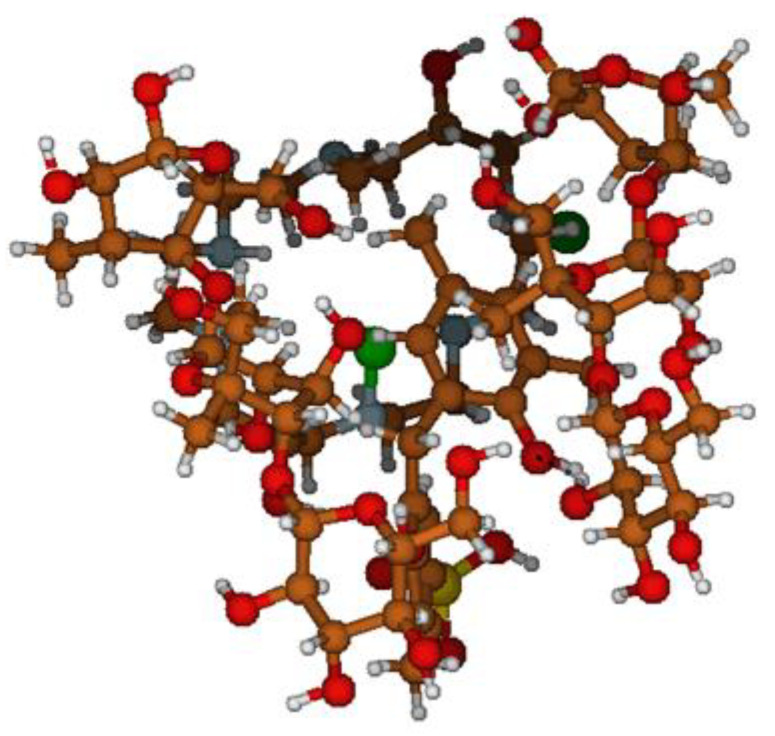
Model of the mosaic membrane Polikon AC.

Using isolated optimized fragments, an elementary molecular model of the developed anion–cation exchange membrane was compiled, containing two elementary microfibril segments localizing anionic and cationic fragments (Figure 5). The optimized minimum possible geometric volume of the obtained molecular model in Å was 16.51 × 16.31 × 19.07, mass 1770.78 amu, total surface area 1818.61 Å^2^, and total structure volume in the van der Waals radius approximation ~1520, 38 Å^3^.

**Figure 6:**
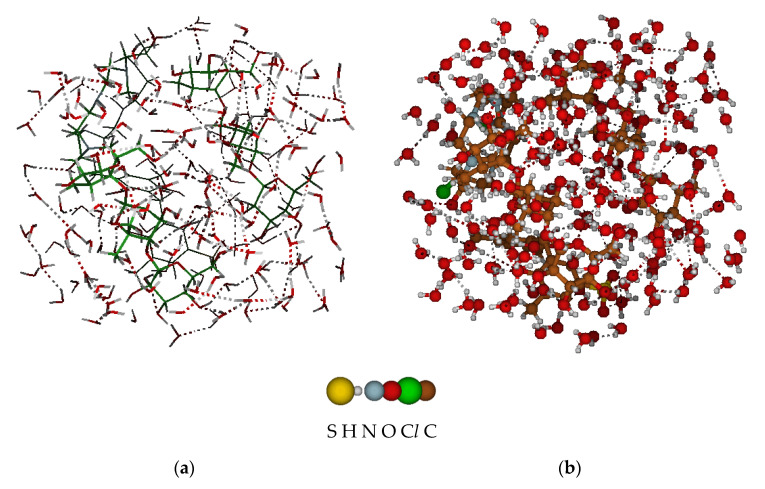
Membrane “Polykon AC” in a water cluster with formed hydrogen bonds (dotted lines). (**a**) A model of the behavior of the “Polikon AC” membrane in an aqueous solution. (**b**) The conformation of the molecular model.

A molecular system consisting of four isolated fragments of the “Polikon AC” mosaic membrane was sequentially placed in a periodic block of a solvation environment of water molecules, which made it possible to implement a model of the behavior of the “Polikon AC” membrane in an aqueous solution (Figure 6a); the total volume of the system was = 4238.58 Å^3^.

A series of statistical, computational experiments made it possible to determine that the effect of a water cluster (~70 water molecules) changes the molecular volume and total surface area of “Polikon AC” within ±2%, with a significant change in the conformation of the molecular model (Figure 6b).

**Figure 7:**
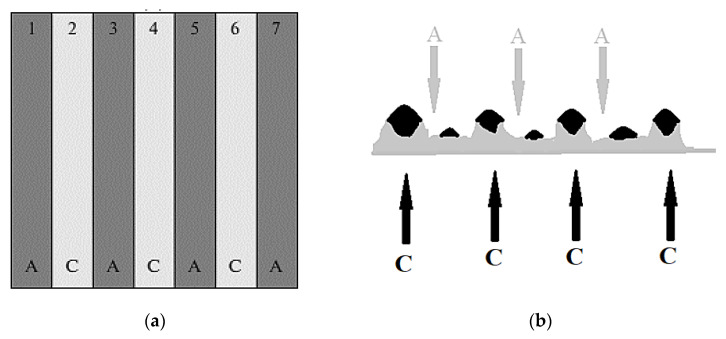
Morphological features of the technology of mosaic membranes “from anion to cation exchanger“. (**a**) Mosaic membranes by the method of polycondensation filling. (**b**) Formation of a cationite matrix. A = anion-exchange, C = cation-exchange.

Experimentally, mosaic membranes “Polikon AC” were obtained on a viscose fiber base by the method of polycondensation filling. At the same time, initially, an anionic matrix was formed, leaving free space on the fiber viscose base for the subsequent formation of a cationite matrix (Figure 7).

The samples obtained were investigated on a TGA Q500 thermogravimetric analyzer and a DMA Q800 dynamic thermomechanical analyzer (TA Instruments, New Castle, DE, USA) in deformation modes, with compression and tension in a temperature range of 20–100 °C.

## 3. Results and Discussion

Fragments of anion and cation exchangers were isolated from the obtained membranes, which were further investigated in dry and wet states. Samples held in deionized water for 24 h have a *w* index. The study was carried out on a dynamic mechanical analyzer, DMA Q800, at a heating rate of 8 °C/min in a temperature range of 25–80 °C and a constant tensile load of 1.5 N.

**Figure 8:**
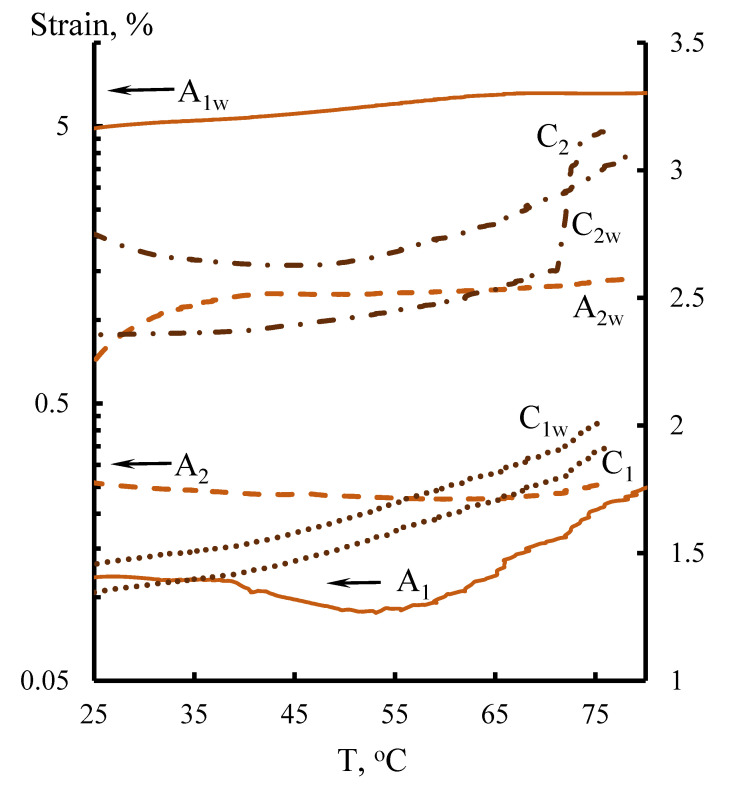
Temperature dependence of deformation for dry and wet (w) anion-exchange = A; cation-exchange = C. Membrane fragments at a constant tensile load of 1.5 N.

Figure 8 shows the temperature dependences of deformation for samples in dry and wet states; it was found that as a result of the polycondensation reaction, a mechanically stable cross-linked composition is formed, strengthened-structured with fragments of a polyfunctional anion exchanger and a bifunctional cation exchanger. Samples A_1_, A_2_, and С_2_ detected states of “glassy” polymer; for the remaining samples, the deformation “of the polymer with a dense mesh structure” manifested. Samples in a wet state had higher values of reversible deformation stability. In the investigated temperature range, constant heating rate and tensile force and the stiffness (in kN/m) of the viscose base samples (in the “dry/wet” states) varied within (1 ÷ 0.79/0.8 ÷ 0.7) anionite (~30/4 ÷ 1.1) and cationite (6 ÷ 4/3.7 ÷ 3.1) fragments.

In the second experiment, the samples were investigated in parallel on two installations simultaneously. The goal was to find the dependence of the deformation on the water content in the cation-exchange and anion-exchange fragments of membranes at a heating rate of 10 °C/min, a maximum temperature of ~100 °C, and a 24-h exposure in deionized water. The tensile load for all samples was the same: 1.5 and 3 N. Upon reaching a maximum temperature of ~100 °C, the samples were kept at an isotherm (similar for a dynamic mechanical analyzer under constant load) for 15 min. The moisture content in the membrane was determined by thermogravimetric curves (Table 1).

**Figure 9:**
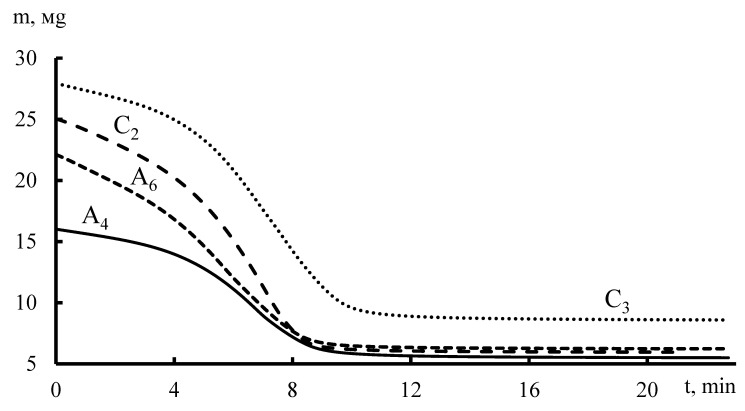
Dynamic change in the mass of wet samples at a heating rate of 10°/min.

According to thermogravimetric analysis data (Figure 9), moisture absorption by membrane fragments was determined. It is shown that the limited moisture (~100%) in the membrane corresponds to the beginning of the research process, the minimum concentration of water in the membrane is at the time when the thermogravimetric curve reaches a plateau, and the mass in this area corresponds to the mass of the dry sample of the membrane.

The moisture content in the samples was determined based on the data in Figure 9 and Table 1, following the expression:

(1) $$m_{H_{2}O}=\frac{m_{i}-m_{dry}}{m_{max}}100\%,$$

where *m_i_* is the mass of the sample at time *t*; *m_dry_* is the mass of a dry sample, a plateau area on the thermogravimetric curve; *m_max_* is the mass of the sample at the moment *t = 0*, the maximum on the thermogravimetric curve.

**Figure 10:**
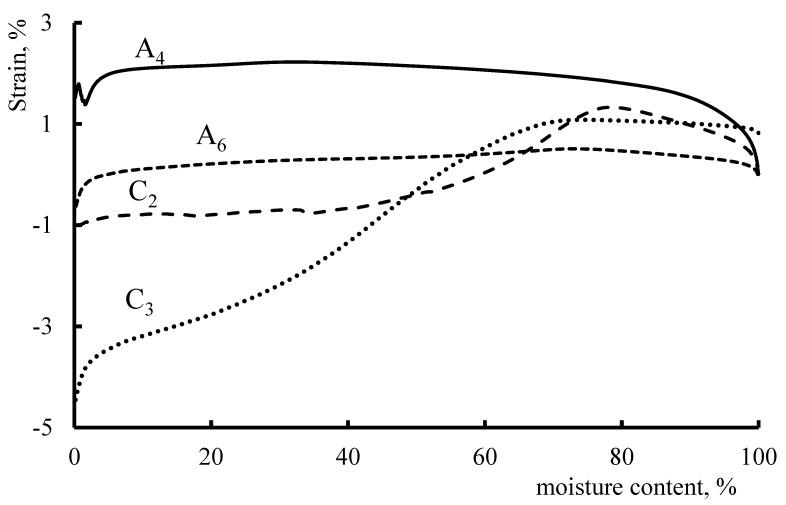
Influence of moisture content on the deformation of anionite and cationite fragments of membranes at a constant external tensile load; N: A_4_–3, A_6_–1.5; C_2_–3, C_3_–1.5.

This enabled us to assess the effect of moisture content on the deformation of anion-exchange and cation-exchange membrane fragments under static external tensile loads (Figure 10).

It has been shown that anionite fragments of membranes are less prone to extreme deformation with an increase in the working temperature and moisture content. Cationite fragments initially show a tendency to “compress”, and with a gradual increase in moisture content and working temperature, stretching begins to prevail, which is explained by the peculiarities of the structures of polymer matrices.

## 4. Conclusions

The success achieved in the development of technology polycondensation filling fibrous matrix monomerization compositions demonstrates the progress and advantages of the method of producing promising composite heterogeneous anion–cation exchange membranes. At the same time, by varying the nature, type, type, and chemical affinity of fibrous substrates to the formed matrices, the industrial production of membranes of the “Polikon AC” type and their effective variable combination in many areas is possible.

This paper presents a refined molecular model membrane “Polikon AC” in a solvation environment. The temperature dependence of the deformation for dry and wet anion-exchange and cation-exchange fragments of membranes under a constant tensile load in a dynamic heating mode was investigated. The effect of moisture content on the deformation of the anionite and cationite fragments of membranes was studied.

The features of the thermomechanical stability of the anion- and cation-exchange matrix “Polikon AC” that we investigated allow us to speak of the effective use of viscose nonwoven materials as a successful fibrous base for the use of a new type of ion-exchange membrane in the processes of fine gas separation and ultrafiltration in such areas as the food industry, antimicrobial dialysis systems, high-tech biomedical units, and pharmaceuticals.

## Figures and Tables

**Table 1:** The moisture content of membrane fragments.

Constant Tensile Load, N	Membrane Fragment	m_max_, mg	m_min_, mg	m_water_, mg
1.5	C_3_	27.94	8.594	19.35
	A_6_	22.11	6.243	15.87
3	C_2_	25.04	5.96	19.09
	A_4_	16.02	5.5	10.51

## Data Availability

Not applicable.
